# Moderate Hypoxia Followed by Reoxygenation Results in Blood-Brain Barrier Breakdown via Oxidative Stress-Dependent Tight-Junction Protein Disruption

**DOI:** 10.1371/journal.pone.0082823

**Published:** 2013-12-06

**Authors:** Christoph M. Zehendner, Laura Librizzi, Jana Hedrich, Nina M. Bauer, Eskedar A. Angamo, Marco de Curtis, Heiko J. Luhmann

**Affiliations:** 1 Institute of Physiology and Pathophysiology, University Medical Center of the Johannes Gutenberg-University, Mainz, Germany; 2 Unit of Experimental Neurophysiology and Epileptology, Fondazione Istituto Neurologico Carlo Besta, Milano, Italy; 3 Centre for Neuroscience and Cell Biology, University of Coimbra, Coimbra, Portugal; Biological Research Centre of the Hungarian Academy of Sciences, Hungary

## Abstract

Re-canalization of cerebral vessels in ischemic stroke is pivotal to rescue dysfunctional brain areas that are exposed to moderate hypoxia within the penumbra from irreversible cell death. Goal of the present study was to evaluate the effect of moderate hypoxia followed by reoxygenation (MHR) on the evolution of reactive oxygen species (ROS) and blood-brain barrier (BBB) integrity in brain endothelial cells (BEC). BBB integrity was assessed in BEC in vitro and in microvessels of the guinea pig whole brain in situ preparation. Probes were exposed to MHR (2 hours 67-70 mmHg O_2_, 3 hours reoxygenation, BEC) or towards occlusion of the arteria cerebri media (MCAO) with or without subsequent reperfusion in the whole brain preparation. In vitro BBB integrity was evaluated using trans-endothelial electrical resistance (TEER) and transwell permeability assays. ROS in BEC were evaluated using 2′,7′-dichlorodihydrofluorescein diacetate (DCF), MitoSox and immunostaining for nitrotyrosine. Tight-junction protein (TJ) integrity in BEC, stainings for nitrotyrosine and FITC-albumin extravasation in the guinea pig brain preparation were assessed by confocal microscopy. Diphenyleneiodonium (DPI) was used to investigate NADPH oxidase dependent ROS evolution and its effect on BBB parameters in BEC. MHR impaired TJ proteins zonula occludens 1 (ZO-1) and claudin 5 (Cl5), decreased TEER, and significantly increased cytosolic ROS in BEC. These events were blocked by the NADPH oxidase inhibitor DPI. MCAO with or without subsequent reoxygenation resulted in extravasation of FITC-albumin and ROS generation in the penumbra region of the guinea pig brain preparation and confirmed BBB damage. BEC integrity may be impaired through ROS in MHR on the level of TJ and the BBB is also functionally impaired in moderate hypoxic conditions followed by reperfusion in a complex guinea pig brain preparation. These findings suggest that the BBB is susceptible towards MHR and that ROS play a key role in this process.

## Introduction

The BBB forms a protective and precisely regulated barrier that separates the central nervous system from peripheral blood circulation[1]. Injuries of the BBB are involved in a number of diseases e.g. ischemic stroke[2] and neuroinflammation[3]. It has been demonstrated that NADPH oxidase has a pivotal role in BBB breakdown in a murine model of MCAO and that the gp91phox (NOX2) containing NADPH oxidase contributes significantly to this process[4]. Further it has been shown in a rat model of stroke that NADPH oxidase activity is significantly elevated in arteries in the penumbra within ischemic brain hemispheres whilst NADPH oxidase dependent superoxide production of the ischemic core is sparse[5]. It has been suggested that the penumbra, the territory at risk for irreversible cell death in ischemic stroke, may be caused by tissue swelling due to early BBB disruption mediated by NADPH oxidase dependent ROS[6]. Recently the brain endothelium has been identified as a major source of ROS production in cerebral ischemia that aggravates cerebral blood flow impairment in reperfusion [7].

These findings indicate that ROS and NADPH oxidase are crucial for BBB pathology in cerebral ischemia. A breakdown of the BBB is associated with brain edema formation[2,8,9] and inflammatory processes that contribute to further cerebral injury. TJ proteins are key structures that ensure the integrity of the BBB[10]. A disruption of TJ goes along with vasogenic brain edema that enhances mortality in ischemic stroke[11]. However it is unclear if the BBB is affected by reoxygenation after MHR and which cellular mechanisms may contribute to BBB perturbances under these circumstances. 

We were therefore interested in the question whether NADPH oxidase in BEC may affect BBB integrity at the level of TJ in moderate hypoxic conditions followed by reperfusion. For this purpose we evaluated

i) the effect of MHR on ROS levels and BBB integrity,
ii) if the NADPH oxidase inhibitor DPI, which is widely used for pharmacological NADPH oxidase inhibition[12], is capable of blocking ROS evolution and BBB impairment during MHR,
iii) BBB integrity after MCAO with or without subsequent reperfusion in the guinea pig brain preparation assessed by intraluminal vs. extravascular FITC-albumin signal.

Apocynin is another widely used inhibitor of the NADPH oxidase, however we preferred DPI in the present investigation as Apocynin may induce ROS[13] in certain cell lines and may not be specific for NADPH oxidase blockade[14]. Recently we have shown that bEnd.3 cells represent a valuable and sensitive model to detect BBB changes in conditions when the interplay of the neurovascular unit is impaired[15]. In brain areas that are subjected towards moderate hypoxia, especially in penumbra conditions, a neurovascular dysfunction is well documented [16]. Therefore we chose the bEnd.3 model to investigate the effect of MHR on BBB integrity. To further strengthen our findings we performed MCAO in the guinea pig whole brain preparation where all cell types of the NVU are preserved except for cellular blood components[17–19]. 

We identified ROS as a key player in BBB injury during MHR at the level of TJ proteins. Our results i) underline the concept that ROS are a hallmark in hypoxic brain injury, ii) demonstrate that the BBB is susceptible towards ROS mediated damage at the level of TJ under moderate hypoxic conditions.

## Results

### Cellular ROS levels but only sparse mitochondrial ROS in brain endothelial cells are elevated in moderate hypoxia followed by reoxygenation

**Figure 1:**
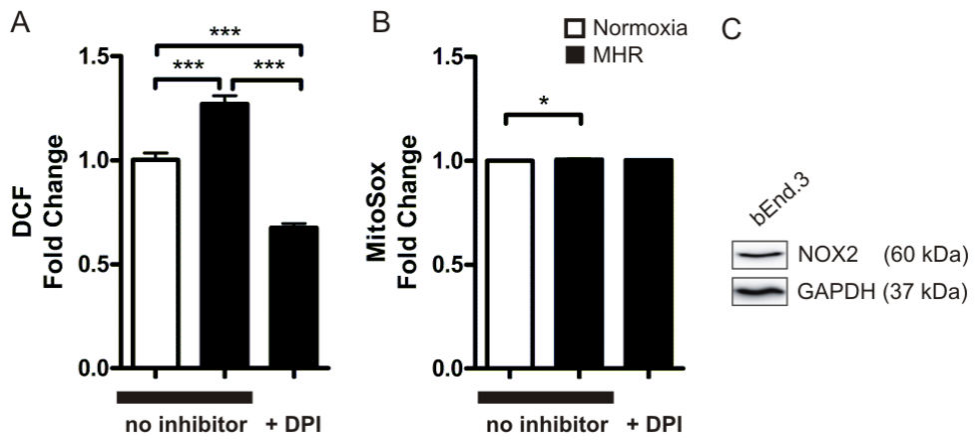
ROS formation in BEC upon MHR and expression of NOX2 in bEnd.3. Significantly elevated ROS levels after MHR were detected using the ROS indicator DCF (normoxia 1 + 0.03 vs. MHR no inhibitor 1.27 + 0.04, P < 0.0001, n = 23 - 48 DCF measurements from 3 - 6 experiments). The NADPH oxidase inhibitor DPI significantly blocked the evolution of ROS (MHR no inhibitor 1.27 + 0.04 vs. MHR+DPI 0.67 + 0.02, P < 0.0001, n = 48 DCF measurements from 6 experiments, A). Mitochondrial ROS levels detected by MitoSox were significantly elevated after MHR but to a lesser extent than ROS detected with DCF (normoxia: 1 + 0.002 vs. MHR 1.01 + 0.003, P = 0.0152). DPI did not block ROS significantly compared with MHR conditions without the inhibitor (MHR no inhibitor 1.01 + 0.003 vs. MHR+DPI 1 + 0.003, P > 0.05, n = 96 MitoSOX measurements from 3 experiments, B, note that in MitoSox measurements SEM is very small so asterisk do not visualize in the graph). Western Blot of bEnd.3 lysates confirmed that bEnd.3 express the NOX2 containing NADPH oxidase.

After MHR cytosolic ROS levels measured with the ROS indicator DCF were significantly higher in solvent treated BEC compared with solvent treated normoxic controls (normoxia 1 + 0.03 vs. MHR 1.27 + 0.04, P < 0.0001, n = 23 - 48 DCF measurements from 3 - 6 experiments, Figure **1 A**). Pre-treatment with the NADPH oxidase inhibitor DPI (DPI concentration was 50 µmol/l in all experiments) reduced ROS levels significantly in MHR compared with solvent treated hypoxic groups (MHR no inhibitor 1.27 + 0.04 vs. MHR+DPI 0.67 + 0.02, P < 0.0001, n = 48 DCF measurements from 6 experiments, Figure **1 A**). DPI also significantly decreased ROS levels in MHR compared with normoxic control (normoxia 1 + 0.03 vs. MHR+DPI 0.67 + 0.02, P < 0.0001, n = 23 - 48 DCF measurements from 3 - 6 experiments). In normoxia DPI significantly lowered ROS levels compared with normoxic controls that were not pre-treated with the inhibitor indicating that DPI lowers basal ROS production in normoxia (normoxia+DPI 0.56 + 0.2 vs. normoxia 1 + 0.03, P < 0.0001, n = 23 ROS measurements from 3 experiments, not depicted in graph). In contrast mitochondrial derived ROS levels were only increased by about 1 % by MHR and DPI pre-treatment had no effect on mitochondrial derived ROS evolution compared with normoxic controls treated with the solvent DMSO (normoxia: 1 + 0.002 vs. MHR no inhibitor 1.01 + 0.003, P = 0.0152; MHR no inhibitor 1.01 + 0.003 vs. MHR+DPI 1 + 0.003, P > 0.05, n = 96 MitoSOX measurements from 3 experiments, Figure **1 B**). In order to verify that bEnd.3 cells express the NOX2 containing NADPH oxidase we performed western blots in BEC in which NOX2 could be detected (Figure **1 C**).

### MHR results in the formation of nitrotyrosine in BEC which is blocked by DPI

**Figure 2:**
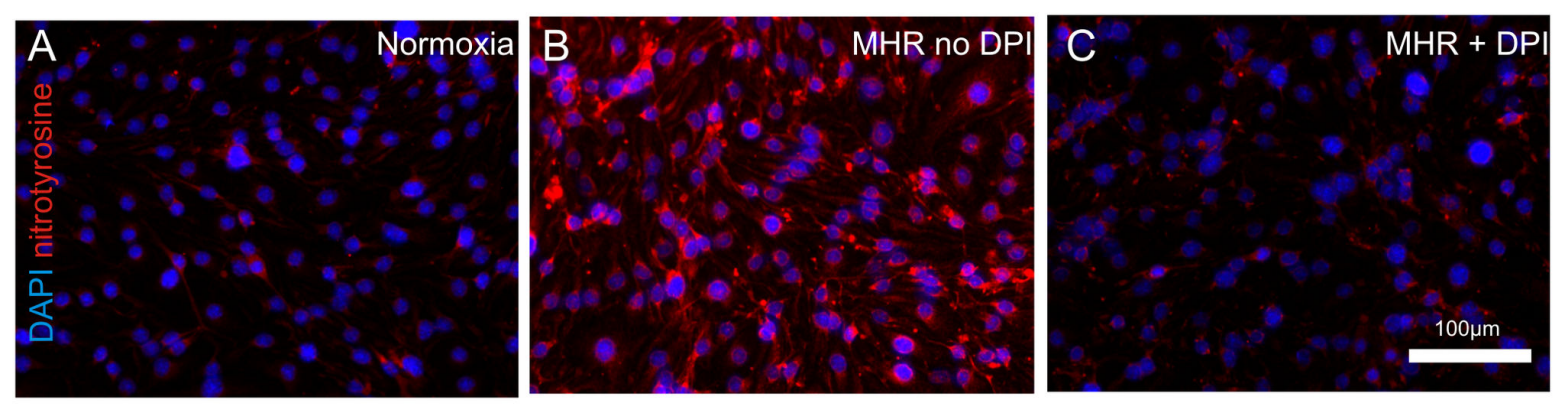
MHR leads to nitrotyrosine formation in BEC that is lowered by DPI. Using immunohistochemical anti-nitrotyrosine stainings we detected only small amounts of nitrotyrosine in solvent treated normoxic controls (**A**). In contrast significantly higher amounts of nitrotyrosine were detected in BEC exposed towards MHR in a cytosolic perinuclear manner (**B**). Pre-treatment with DPI blocked nitrotyrosine formation after MHR (**C**). Representative images from 3 experiments are shown.

Nitrotyrosine is formed by the reaction of the amino acid tyrosine and the ROS peroxynitrite and is therefore an indicator for oxidative stress[7]. To strengthen the significance of our finding that cellular ROS are elevated upon MHR in BEC we performed nitrotyrosine stainings in bEnd.3 cells after MHR. The sensitivity of the nitrotyrosine antibody was confirmed by using peroxynitrite as a positive control as recommended by the manufacturer. We found that nitrotyrosine formation in BEC was higher than in normoxic controls. In addition pre-treatment with DPI abolished nitrotyrosine formation (representative images from 3 experiments, Figure **2**) 

### Trans-endothelial electrical resistance (TEER) is reduced by MHR in an oxidative stress dependent manner

**Figure 3:**
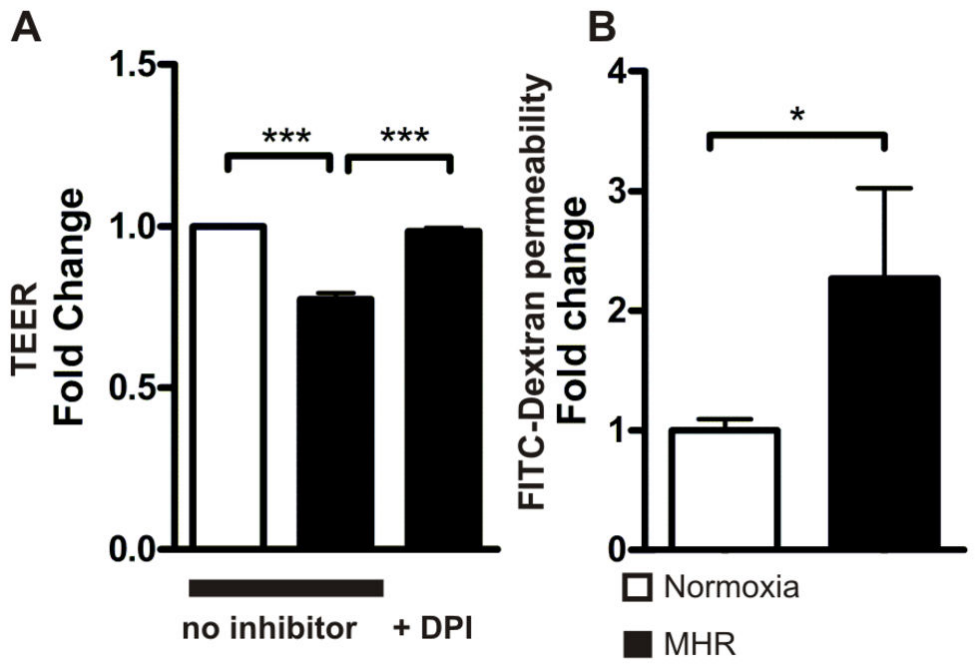
MHR impairs TEER in Brain endothelial cells and increases transcellular permeability towards high molecular mass molecules. Trans-endothelial resistance values detected with ECIS (**A**) were significantly lower in BEC after MHR than in normoxic conditions (normoxia 1 + 0.004 vs. MHR no inhibitor 0.77 + 0.02, P < 0.0001; n = 169 - 288 TEER measurements from 3 experiments). DPI prevented the decrease of TEER significantly (MHR+DPI 0.98 + 0.01 vs. MHR no inhibitor 0.77 + 0.02, P < 0.0001; n = 168 - 192 TEER measurements from 3 experiments). MHR resulted in an increase of FITC-Dextran (150,000 Dalton) permeability in bEnd.3 monolayers (normoxia 1 + 0.09 vs. MHR 2.27 + 0.75, P < 0.05; n = 7-15 permeability assays, **B**).

To quantify BBB impairment upon MHR we performed TEER measurements. MHR resulted in significantly lower TEER values in hypoxic groups compared with normoxic controls (normoxia 1 + 0.004 vs. MHR no inhibitor 0.77 + 0.02, P < 0.0001; n = 169 - 288 TEER measurements from 3 experiments). To address the question if reactive oxygen species are involved in the detected TEER decrease after MHR we applied the NADPH oxidase inhibitor DPI ahead of MHR which was found to decrease ROS levels in our experimental setting. Here, TEER values were significantly higher than in solvent treated cells exposed towards MHR (MHR+DPI 0.98 + 0.01 vs. MHR no inhibitor 0.77 + 0.02, P < 0.0001; n = 168 - 192 TEER measurements from 3 experiments, Figure **3 A**). DPI has been reported also to also inhibit nitric oxide synthases (NOS) that produce the reaction product NO[20] and may also inhibit the xanthine oxidase (XO)[21]. To elucidate if NO blockade or XO inhibition may have a similar protective effect on TEER as DPI we treated bEnd.3 cells with the NOS inhibitor N_ω_-Methyl-L-arginine acetate (LNMMA, 300 µmol/l)[20] and the XO inhibitor allopurinol 125 µmol/l [22]. We chose these concentrations because they have been reported to have a significant biological effect on endothelial cells[23–25]. Here TEER values were highly significantly lower compared with probes that were treated with DPI (MHR+DPI 0.98 + 0.01 vs. MHR+LNMMA 0.7 + 0.02, P < 0.001; MHR+DPI 0.98 + 0.01 vs. MHR+allopurinol 0.65 + 0.02, P < 0.001; n = 73 - 192 TEER measurements per condition from 3 independent experiments, not depicted in graph). 

### MHR induces increased trans-endothelial permeability to macromolecules

Under physiological conditions the BBB is impermeable to macromolecules circulating in the blood stream, e.g. albumin (mass weight around 70,000 Dalton)[26]. In order to evaluate if the detected decrease in TEER upon MHR is also reflected by an enhanced permeability level of BEC monolayers towards macromolecules we performed permeability assays with fluorescein isothiocyanate–dextran (FITC-Dextran, mass weight 150,000 Dalton). We found that MHR results in a significant increase in FITC-Dextran permeability compared with normoxic controls (normoxia 1 + 0.09 vs. MHR 2.27 + 0.75, P < 0.05; n = 7-15 permeability assays, Figure **3 B**, relative permeability values are displayed). FITC-Dextran permeability in DPI treated BEC was not significantly elevated compared with normoxic controls (relative permeability values: normoxia 1 + 0.09 vs. MHR+DPI 1.5 + 0.3, P > 0.05; n = 7-15 permeability assays, not shown in graph).

### Oxidative stress in MHR affects tight-junction protein expression of ZO-1 and Claudin 5 on bEnd.3 cell membranes

**Figure 4:**
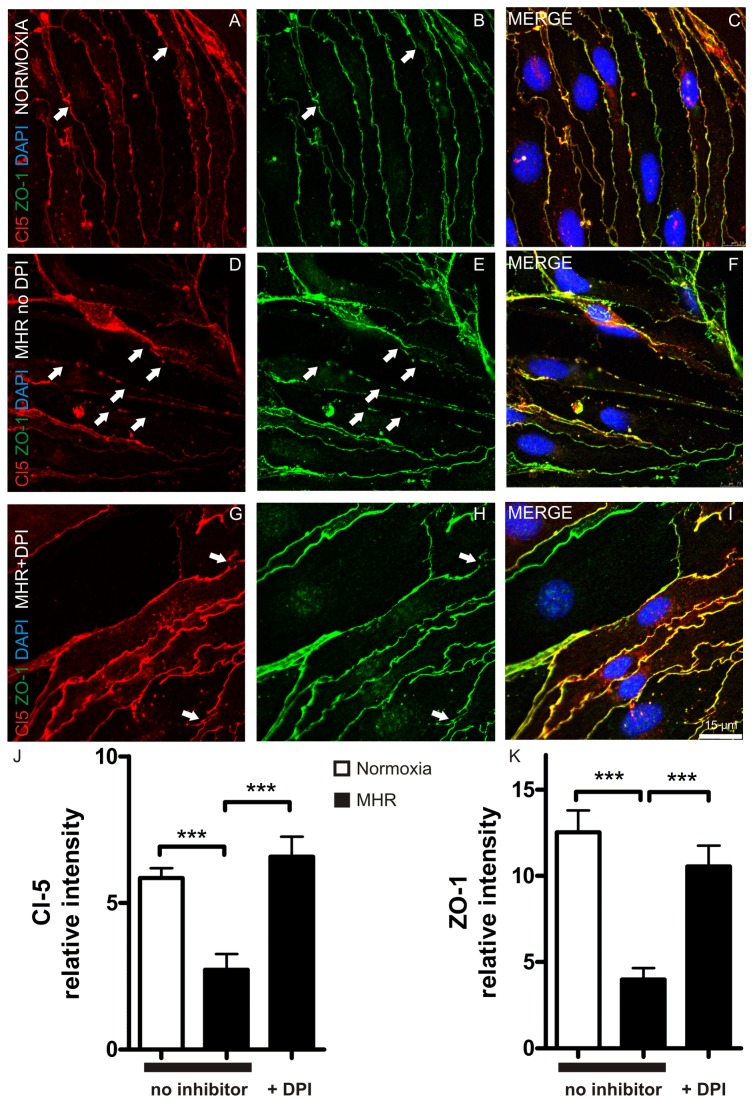
MHR disrupts BEC integrity on the level of tight junction proteins. BEC displayed a physiological arrangement of TJ proteins Claudin 5 (**A**) and zonula occludens 1 (**B**) in a co-localizing manner (**C**) with only few TJ gaps (arrowheads). MHR disrupted the continuous arrangement of both junctional proteins in BEC (arrowheads **D-F**). Here, DPI blocked TJ injury in MHR, only few TJ disruptions were detected in BEC pre-treated with the NADPH oxidase inhibitor DPI (arrowheads **G-I**). Representative images from 3 experiments per group are shown. Quantitative analyses of Cl5 (Cl5 normoxia: 5.85 + 0.33 vs. Cl5 MHR: 2.72 + 0.54, P < 0.0001; n = 12 cells per group from 3 experiments, **J**) and ZO-1 (ZO-1 normoxia: 12.53 + 1.27 vs. ZO-1 MHR: 3.98 + 0.67, P < 0.0001; n = 12 cells per group from 3 experiments, **K**) integrity on cell membranes revealed that both junctional proteins are significantly perturbed in MHR. DPI pre-treatment significantly restored TJ integrity at cellular membranes under MHR (Cl5 MHR: 2.72 + 0.54 vs. Cl5 MHR+DPI: 6.57 + 0.69, P = 0.0002; ZO-1 MHR: 3.98 + 0.67 vs. ZO-1 MHR+DPI 10.55 + 1.21, P < 0.0001; n = 12 cells per group from 3 experiments).

In the next step we sought to identify the cellular mechanism for TEER decrease and increase of permeability of BEC monolayers in MHR. We have previously shown that TJ integrity correlates with TEER in BBB models in vitro[15,27]. They are hallmarks for BBB integrity and are of major importance for BBB stability in hypoxic and other pathologic conditions[3,9,28]. Therefore we were interested in the question if the TJ proteins ZO-1 or Cl5 are involved in MHR mediated BBB injury. Immunohistochemical stainings in BEC monolayers demonstrated that both TJ proteins are disrupted in MHR compared with intact ZO-1 and Cl5 arrangement in normoxic controls (representative images from 3 experiments, Figure **4 A - F**). In the next step we wanted to find out if the previously observed protective effect of DPI on TEER is also reflected by a preservation of TJ alignment on cell membranes through DPI pre-treatment in MHR. In MHR DPI resulted in a preserved expression of ZO-1 and Cl5 on cell membranes compared with DMSO treated MHR probes (Figure **4 D-I**, representative image from 3 experiments). Quantitative analyses of TJ integrity on cell membranes confirmed these findings (Cl5 normoxia: 5.85 + 0.33 vs. Cl5 MHR no inhibitor: 2.72 + 0.54, P < 0.0001; n = 12 cells per group from 3 experiments, Figure **4 J**; ZO-1 normoxia: 12.53 + 1.27 vs. ZO-1 MHR no inhibitor: 3.98 + 0.67, P < 0.0001; n = 12 cells per group from 3 experiments, Figure **4 K**). DPI abolished Cl5 (Cl5 MHR no inhibitor: 2.72 + 0.54 vs. Cl5 MHR+DPI: 6.57 + 0.69, P = 0.0002; n = 12 cells per group from 3 experiments, Figure **4 J**) and ZO-1 (ZO-1 MHR no inhibitor: 3.98 + 0.67 vs. ZO-1 MHR+DPI 10.55 + 1.21, P < 0.0001; n = 12 cells per group from 3 experiments, Figure **4 K**) impairment in MHR.

### BBB impairment within the penumbra region in MCAO

**Figure 5:**
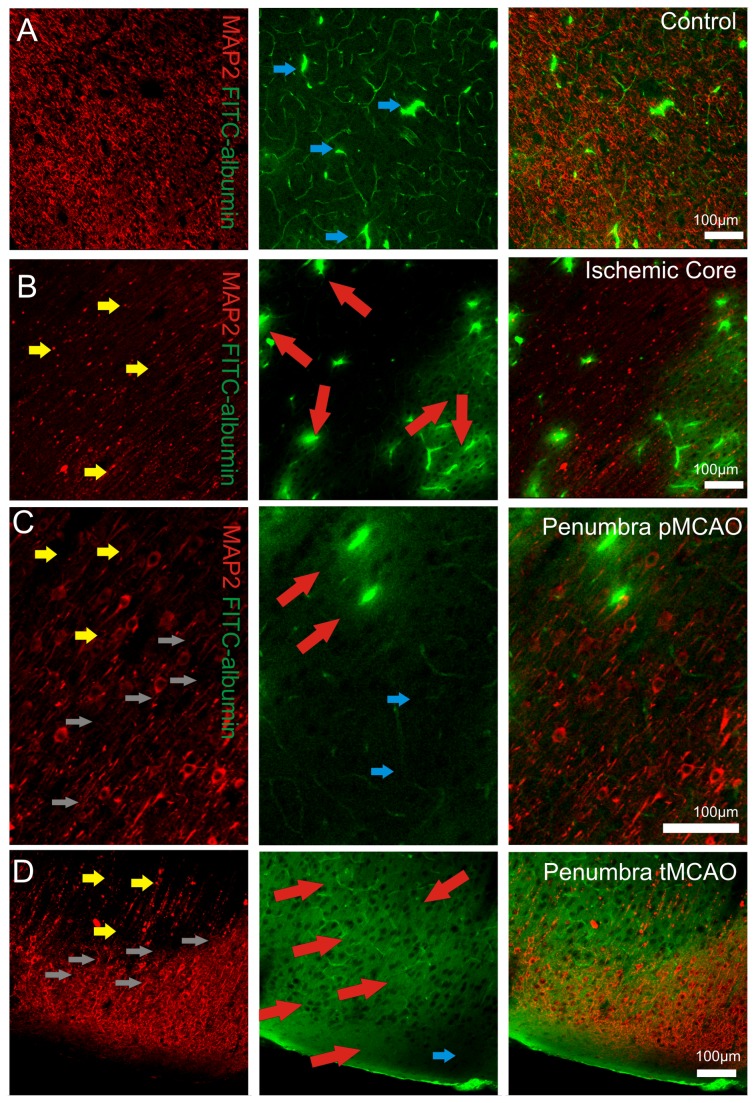
FITC extravasation in microvessels after MCAO. Representative confocal fluorescence images of MAP 2 (red), Fluorescein isothiocyanate –albumin (FITC-albumin; green) in the neocortex of pMCAO n = 3 brains and tMCAO n = 8 brains are depicted. Blue arrows mark intact regions of BBB that partly contain intraluminal entrapped FITC-albumin (note that no green halos are present around the vessels), yellow arrows indicate MAP 2 impairment, red arrows indicate FITC-albumin extravasation. In control hemispheres (contralateral to MCAO hemispheres) MAP 2 immunoreactivity was unaffected and FITC-albumin signals were found to be situated intraluminal (blu arrows, **A**). The ischemic core was characterized by a markedly reduced MAP 2 signal (yellow arrows, **B**) and areas of parenchymal FITC–albumin extravasation (red arrows) around vessels. In the penumbra transition zone of permanent MCAO (grey arrows, **C**) FITC-albumin extravasation (red arrows) was found to be present in areas where MAP2 signals were reduced (yellow arrows, **C**). In the penumbra transition zone (grey arrows, **D**) during transient MCAO followed by reperfusion we found a wide and diffuse parenchymal FITC–albumin extravasation (red arrows, **D**) that extended into areas with intact MAP2 immunoreactivity. The blue arrow marks the region where no FITC-albumin extravasation was found.

To strengthen the significance of our in vitro findings that BBB integrity is impaired by MHR we performed MCAO occlusion with or without subsequent reperfusion in the isolated guinea pig brain preparation. Contralateral perfused hemispheres served as controls for each MCAO setting. The extravasation of FITC-albumin in the penumbra region was assessed. Albumin is a macromolecule that does not cross the BBB in physiological states[29]. In both conditions perfused control hemispheres were characterized by an intact MAP2 immunoreactivity in conjunction with intact brain microvessels that partly displayed entrapped intravascular FITC-albumin (Figure **5 A**). Loss of MAP2 signal is an early marker for dendritic damage[30] and has been already used to identify the penumbra region in focal ischemia in the guinea pig brain preparation[31]. During permanent MCAO (pMCAO 1.5 hours without reperfusion, n = 3 brains) perivascular leakage of FITC-albumin and a heavily impaired MAP 2 immunoreactivity in the ischemic core was observed (Figure **5 B**). With the help of MAP 2 staining we identified the penumbra[31] zone where we also detected FITC-albumin extravasation (Figure **5 C**). To find out if reperfusion also induces BBB impairment in the penumbra zone after reperfusion a set of experiments in which MCAO was followed by 1.5 hours of reperfusion (tMCAO, n = 8 brains) was performed. In this condition large and diffuse extravasation of FITC-albumin became apparent that extended into areas of intact MAP 2 immunoreactivity (Figure **5 D**). 

### Formation of ROS in cerebral microvessels in the penumbra region after MCAO followed by reperfusion

**Figure 6:**
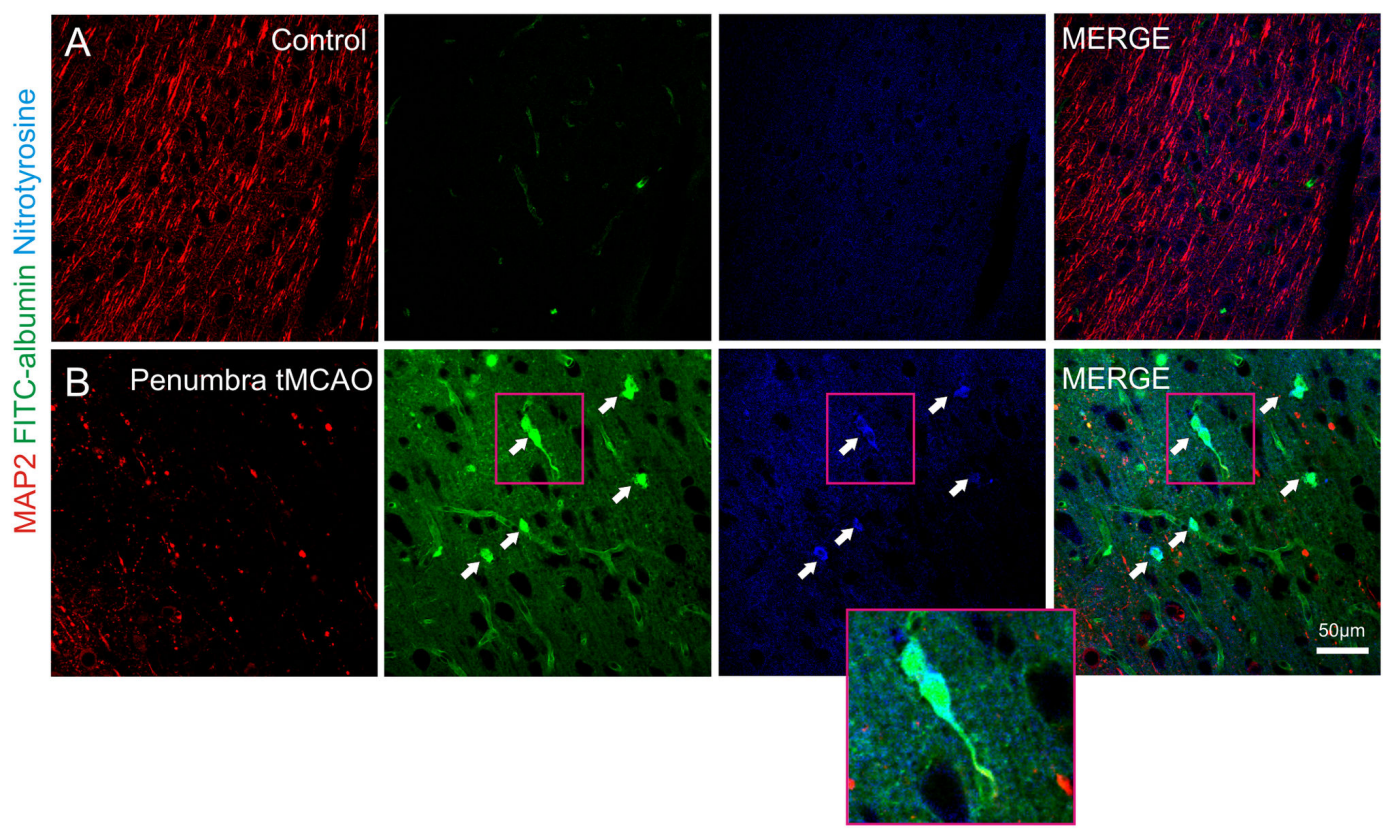
MCAO followed by reperfusion leads to the generation of ROS in cerebral microvessels. Images from a representative control (**A**) hemisphere and its contralateral tMCAO hemisphere (**B**) of the neocortex are shown. MAP 2 (red), Fluorescein isothiocyanate –albumin (FITC-albumin; green) and anti-nitrotyrosine (blue) are displayed (n = 3 brains). Perfused control hemispheres (contralateral to tMCAO) displayed an intact MAP2 immunoreactivity, no FITC-albumin extravasation and absence of nitrotyrosine formation (**A**). In the penumbra of corresponding tMCAO hemispheres MAP2 damage was present as well as large and diffuse FITC-albumin extravasation. Here we found that cerebral microvessels (indicated by arrows and inset) were immunoreactive for nitrotyrosine indicating the formation of ROS (**B**). Note the cytosolic staining pattern of nitrotyrosine indicating the cytosolic localization of ROS.

Next we were interested in the question if ROS were also generated in cerebral microvessels of the penumbra region in the setting of tMCAO where diffuse and large FITC-albumin extravasation occurred. Perfused contralateral control hemispheres displayed an intact MAP2 immunoreactivity with lack of FITC-albumin extravasation. Here immunoreactivtiy for nitrotyrosine was absent (Figure **6 A**). In contrast we found nitrotyrosine formation in cerebral microvessels within the penumbra region of hemispheres that underwent MCAO followed by reperfusion (Figure **6 B**). Representative images from 3 experiments are displayed.

## Discussion

In the present study we show that ROS have the capability to impair BBB integrity in moderate hypoxia followed by reoxygenation. The main findings of our study are i) MHR results in a significant increase of cellular ROS in BEC which can be lowered by the NADPH oxidase blocker DPI. ii) MHR impairs BEC at the level of TEER, increases BEC monolayer permeability towards macromolecules and disrupts TJ proteins ZO-1 and Cl5 in an oxidative stress dependent manner. iii) FITC-albumin extravasation occurs in the penumbra zone in MCAO conditions with or without subsequent reperfusion in the guinea pig whole brain in situ preparation.

ROS are a hallmark in ischemic brain injury[8,32,33]. Especially brain swelling in ischemic stroke is of high clinical relevance in patients suffering brain ischemia[11]. It has been postulated that ROS directly contribute to the evolution of the penumbra[6], an area in the brain subjected towards moderate hypoxia with perturbed function of evoked responses, impaired extracellular potassium levels[16] and risk for irreversible cell death[34]. This area can be potentially rescued by reperfusion if reoxygenation is restored within a few hours after onset of clinical symptoms[35]. After passing this “window of opportunity” clinical benefit of reperfusion therapy decreases due to the risk of intracranial bleeding and other unwanted side effects of reperfusion therapy[36]. Therefore approaches are made to extend this time window of thrombolytic therapy[37]. However no effective treatment strategy has yet been established to treat ischemic stroke if reperfusion is delayed. Hence mechanisms that destabilize BBB integrity during reperfusion of moderate hypoxic areas in the penumbra are desirable to unravel better treatment strategies in ischemic stroke. 

In our experimental design MHR we lowered oxygen levels to 70 mmHg (which is a reduction of about ~ 50-55% of normoxic controls) because this relative reduction in pO_2_ mimics the relative pO_2_ decrease that is present within the penumbra in a rat stroke model[38]. Our results on ROS formation in bEnd.3 after MHR in vitro show that ROS are mainly generated within the cytosol of BEC. This rise in ROS levels in ischemic BEC is in line with observations from previous work in our laboratory[39] as well as the work of other groups that used more severe hypoxic conditions[40]. Interestingly the level of DCF increase in the present study after MHR was similar to DCF levels observed in a more severe setting of hypoxia followed by reoxygenation in a coculture model of the BBB[39]. In order to determine the pO_2_ threshold which is necessary to induce ROS in BEC more future experimental work is needed. Our data is further congruent with results from Miller and colleagues who reported an elevated activity of NADPH oxidase in cerebral arteries within the penumbra in an in vivo MCAO study in rats that was performed by application of endothelin[5]. However the cellular mechanism of activation of the NADPH oxidase in our experimental setting is unclear. It has been reported in experiments from lung endothelium that an increase of potassium levels, which occurs e.g. during cell death, may lead to gp91phox activation and induces ROS in a similar manner as ischemia[41]. Therefore changes in potassium levels may be a possible pathway of NADPH oxidase activation[41,42] in MHR but clearly more experiments are needed to address this question in detail. 

To our knowledge we are the first to show that a significant amount of ROS is produced in moderate hypoxia at a pO_2_ of about 70 mmHg followed by reoxygenation in BEC in vitro. Further this finding was underlined by the formation of nitrotyrosine (which is another indicator for ROS) in bEnd.3 cells following MHR that was also present in impaired cerebral microvessels in the penumbra in tMCAO. Yemisci and colleagues have shown that reperfusion of brain areas after MCAO is hindered due to peroxynitrite-mediated vasoconstriction of cerebral microvessels by pericytes[7]. Our results on nitrotyrosine formation in MHR and tMCAO point out that the vasoconstrictor peroxynitrite may already evolve during moderate hypoxic states. Thereby brain circulation could be already affected significantly in moderate hypoxic conditions. 

However more detailed investigations regarding the effect of MHR on pericyte pathophysiology will be required to fully address this question. Interestingly we detected a significant but sparse increase of mitochondrial derived ROS in MHR. It has been speculated that mitochondrial ROS are another major source for BBB impairment in ischemia followed by reperfusion[43]. However the authors of that experimental study induced ischemia via chemical hypoxia which is a different experimental condition than in our setup. Because the increase of mitochondrial ROS was only about 1%, we speculate mitochondria are not the major source for ROS generation in MHR. The protective effects exerted by DPI on TEER, TJ protein integrity, decrease of ROS and nitrotyrosine point to a significant contribution of NADPH oxidase to BBB injury in MHR in bEnd.3 cells. This conclusion is further supported by the finding that the NO inhibitor LNMMA and the XO inhibitor allopurinol did not have a similar protective effect on TEER like DPI. This is important to note because it has been reported that DPI may also act on NOS and the XO[21]. In our setting DPI also significantly reduced ROS levels compared with normoxic controls. This finding could be explained by the fact that a certain level of basal ROS is produced by the NADPH oxidase under physiological conditions[5,44,45]. This is supported by our finding that DPI also lowered DCF signals significantly in normoxic conditions compared with untreated normoxic controls. In our study design we pre-treated bEnd.3 ahead of MHR. One may argue that this protective effect may not be of clinical relevance because pre-treatment in stroke is practically not feasible. However ischemic stroke is a highly dynamic process. Brain perfusion changes over time with the onset of stroke[46], tissue swelling affects cerebral microcirculation in a time dependent manner and thereby brain perfusion diminishes in the affected ischemic brain territory[47]. With onset of clinical symptoms it is likely that brain areas that are initially still perfused, and thereby targetable with e.g. intravenous administered drugs, may be subjected towards moderate hypoxia conditions in the course of the ischemic event. We therefore speculate that in ischemic stroke it may be beneficial to apply drugs that stabilize BBB at the timepoint when clinically focal deficits become apparent. At this timepoint therapeutics will very likely reach brain areas that are at high risk of being exposed towards MHR in the course of the disease. Importantly we observed a protective effect induced by the NADPH oxidase inhibitor DPI on BEC. In a clinical setting this would be of high interest because drugs targeting solely the brain endothelium, and not the brain parenchyma, would not need to bypass the BBB.

MHR conditions are of high relevance to mimic penumbra-like conditions[38]. Because in our bEnd.3 in vitro model some components of the neurovascular unit are lacking we further analyzed BBB integrity in the ischemic core and penumbra zone of the guinea pig whole brain in situ preparation after MCAO followed or not by reperfusion. This model better reflects the in vivo situation because the structural and functional preservation of the neurovascular compartments as well as the BBB is maintained except for cellular blood components[17–19]. 

Of note we found a wide BBB impairment in the penumbra zone reflected by FITC-albumin extravasation that was pronounced in those experiments in which artery occlusions was followed by reperfusion. Our results therefore indicate that in those areas affected by moderate ischemia, the BBB remains impaired despite the reopening for reoxygenation of the occluded vessel in the guinea pig whole brain preparation. Findings by Bauer and colleagues who have shown that prolonged moderate hypoxia (8% normobaric O_2_ atmosphere for 2 days) in mice results in brain edema formation which is caused by ZO-1 and occludin impairment also support our conclusion that moderate hypoxic states significantly impair BBB integrity. As Yemisci et al. already nicely demonstrated reopening of the occluded vessel in MCAO may not result in a successful reperfusion due to pericyte constriction[7]. However in the presented in vitro conditions we also observed a BBB damage after MHR represented by decreased values of TEER, a sensitive tool to measure BBB integrity[48]. In addition MHR also increased permeability of BEC monolayers towards high molecular mass FITC-Dextran. In both experimental conditions reoxygenation is ensured by a normoxic incubator and cannot be influenced by microvascular constrictions. We therefore suggest that BBB impairment is mediated by ROS that result in cellular impairment of TJ proteins which cannot be fully prevented even if reoxygenation is successfully performed. Here we would like to stress a limitation of our study: all mechanistic investigations regarding DPI and readouts on TEER, FITC-Dextran, ZO-1 and Cl5 in this presented report were performed in the in vitro bEnd.3 model only, whilst the in situ guinea pig whole brain preparation was used to confirm functional BBB damage by FITC-albumin leakage and ROS generation in the penumbra zone in the setting of tMCAO.

TJ proteins have a fundamental role in maintaining BBB integrity during various pathological conditions[3,10,49] and we have previously shown that Cl5 and ZO-1 are highly susceptible towards ischemic events in RANUD (Rapid Anoxic Neurovascular Unit Damage)[50]. Based on the results of the present study we suggest that impairment of Cl5 and ZO-1 also has a fundamental role in BBB injury in MHR conditions. We previously showed that Cl5 and ZO-1 integrity correlate with TEER in BEC[27,50]. In addition we also show that MHR not only affects TEER and TJ but also increases macromolecular permeability of BEC monolayers. However it has been documented that persistence of Cl5 may also have adverse effects in brain trauma in an in vivo study in mice[51]. Here targeted suppression of Cl5 improved outcome by reducing cerebral edema which is somewhat in contrast to the literature[10]. This underlines that TJ pathophysiology may vary depending on the pathophysiological stimulus. Here we would like to emphasize that we cannot exclude that the observed BBB impairment by MHR may be reversible. However TJ are not only involved in making the BBB impermeable to macromolecules but they are also pivotal in neuroinflammation. It has been noted that neuroinflammation following ischemic stroke in mice significantly worsens brain damage [52]. In cerebral inflammation TJ proteins have a pivotal role (for a detailed review see Coisne and Engelhardt) [3].

We therefore propose that a major cellular mechanism of BBB damage in MHR is represented by TJ protein impairment through ROS. We speculate that this finding may not only be of relevance for ischemic stroke but may also have implications in understanding disorders of the CNS that go along with moderate hypoxia e.g. vascular dementia and may be linked with neuroinflammation. 

## Materials and Methods

### Cell culture

The murine brain endothelial cell line bEnd.3 (passages 12-28; from American Type Culture Collection, Manassas, Virginia, USA) was cultured as recommended by the manufacturer. Cells were kept at 37°C, 5% CO_2_ in a humidified atmosphere. bEnd.3 medium was composed of DMEM Glutamax with 2% Penicillin/Streptomycin (all from Invitrogen GmbH, Karlsruhe, Germany) and fetal calf serum 15% (Biochrom AG , Berlin, Germany). All inhibitors were pre-incubated for 60 minutes ahead of induction of moderate hypoxia. Normoxic and MHR groups that did not undergo pre-treatment with inhibitors were treated with the solvent DMSO in which inhibitors were dissolved (final DMSO amount < 0.01%).

### Guinea Pig whole brain preparation

The experimental protocol was reviewed and approved by the Committee on Animal Care and Use and by the Ethics Committee of the Fondazione Istituto Neurologico “C. Besta”, in accordance with national and international guidelines on care and use of laboratory animals (protocol 45–46; 07/11/2003). All efforts were made to minimize the number of guinea pigs used and their suffering. For experiments brains of young adult Hartley guinea pigs were used (150-200 g body weight; obtained from Charles River, Calco, Italy). Following anaesthesia with intraperitoneal injection of sodium thiopental (80 mg/kg Farmotal, Pharmacia,Milan), a transcardiac perfusion was performed with a cold oxygenated saline solution (composition see below; pH 7.1). The brain was isolated in vitro following the standard technique described elsewhere[17,53]. Briefly, after anaesthesia the brain was carefully isolated and transferred to an incubation chamber. A polyethylene cannula was inserted in the basilar artery to ensure arterial perfusion with a saline solution (composition: NaCl, 126 mM, KCl,3 mM, KH2PO4, 1.2 mM, MgSO4, 1.3 mM, CaCl2, 2.4 mM, NaHCO3, 26 mM, glucose, 15 mM, 3% dextran M.W.70000), oxygenated with a 95%O_2_-5%CO_2_ gas mixture (pH 7.3). Arterial perfusion at 7 ml/min was carried out via a perfusion pump (Gilson Minipulse, France). Brain isolation was performed at low temperature (15°C) and experiments were carried out at 32°C. In these conditions the brains maintain their physiological properties for several hours[17–19,53,54]. In all image acquisitions of the guinea pig analyses images were acquired in the same conditions in terms of gain, laser intensity, pinhole size. Background subtraction was zero.

### Middle cerebral artery occlusion and reperfusion

To induce focal ischemia, the proximal portion of one of the MCA was isolated from the surrounding dura and a loose silk thread node was placed around the vessel. Then the extremities of the node threads were pulled and the MCA was occluded for 1.5 hours. In those experiments in which reperfusion was performed the node was released to allow reperfusion of the isolated brain after 1.5 hours of MCAO. To assess the extent of BBB breakdown fluoresceinisothiocyanate-albumin (FITC–albumin, 50mg/10ml, Sigma-Aldrich) was perfused for 5 minutes at the end of the experiments.

### Moderate hypoxia followed by reoxygenation

MHR was induced by switching the culture medium to HBSS (2 mmol/l CaCl_2_, 1 mmol/l MgCl_2_). Medium was bubbled with a gas mixture of 8% O_2_, 5% CO_2_, 87% N_2_ and after an equilibration period of 20 minutes at 37°C, 8% O_2_, 5% CO_2_, 87% N_2_ in a humidified atmosphere probes were kept for another 2 h in a C42 incubator equipped with CO_2_ and O_2_ sensors at 37°C, 8% O_2_, 5% CO_2_, 87% N_2_ (Labotect, Göttingen, Germany). Normoxic controls which were treated with the vehicle DMSO also underwent a medium exchange with HBSS (2 mmol/l CaCl_2_, 1 mmol/l MgCl_2,_ 10 mmol/l glucose; HBSS^+++^) that was equilibrated to 20% O_2_, 5% CO_2_, 75% N_2_. After 2h probes were reoxygenated for 3 hours (37°C, 20% O_2_, 5% CO_2_, 75% N_2_, humidified atmosphere) in HBSS^+++^. To assess the efficacy of MHR pO_2_ values in mmHg were determined at the beginning (MHR1) and after 2 hours of MHR (MHR2) with an oxygen sensing probe (Oxylite, Oxford Optronix). pO_2_ values were significantly lower in our MHR protocol than in control media (MHR1: 67.97 + 0.17 mmHg vs. Control 128.1 + 0.01 mmHg, P < 0.0001; MHR2: 69.26 + 0.34 mmHg vs. Control 128.1 + 0.01 mmHg, P < 0.0001, n = 3 per group).

All inhibitors used in this study were pre-incubated for 60 minutes in culture medium ahead of induction of MHR. 

### Dyes, chemicals

FITC-Dextran (1 mg/ml, mass weight 150,000 Dalton), allopurinol (125 µmol/l), N_ω_-Methyl-L-arginine acetate salt, (L-NMMA 300 µmol/l), DAPI (0.5 µg/ml) and triton X-100 were from Sigma-Aldrich (Steinheim, Germany), diphenyleneiodonium (DPI; 50 µmol/l, we chose this concentration because it has been successfully used by Hansel et al. to block NADPH oxidase[12]), 2′,7′-Dichlorodihydrofluorescein Diacetate (DCF; 10 µmol/l) (all from Calbiochem, Darmstadt, Germany), HBSS, MitoSOX Red (MitoSox, 5 µmol/l), Cell trace calcein red AM (calcein red, 5µmol/l), Cell trace calcein green AM (calcein green, 5µmol/l) were from Invitrogen GmbH, Karlsruhe, Germany. CaCl_2_ was from Riedel-de Haën (Seelze, Germany), MgCl_2_ was from Merck, Darmstadt, Germany, Fluoromount was purchased from Southern Biotech (Birmingham, USA). 

### Evaluation of electrical resistance

For evaluation of TEER bEnd.3 were seeded at a density of 40,000 cells/well in 96 W Electrical Cell Impedance Sensing arrays (ibidi in cooperation with Applied BioPhysics, Martinsried, Germany). When wells reached maximal impedance values moderate ischemia was induced as described above. Only cells that reached an impedance of at least 600 Ohm were further used in the course of the experiments. On average bEnd.3 cells reached plateau values of 969 + 117 Ohm (+standard deviation), growth area 0.3 cm^2^ -> 291 + 35 Ohm X cm^2^. TEER values obtained after experimental manipulation (MHR / normoxic control conditions) were set into relation to TEER values before MHR / normoxic control induction. Relative changes of electrical resistance measurements which were related to solvent (DMSO treated) controls are displayed. 

### ROS detection

bEnd.3 (40,000 cells/well) cells were seeded in 96 Well black Greiner culture flasks. bEnd.3 were grown to confluence within 48 hours. Confluence was evaluated ahead of each experiment via light microscopy. The absolute amount of ROS production is dependent on viable cell numbers because ROS are generated by e.g. mitochondria[55,56] or enzymes like the NADPH oxidase within living cells[57]. Hypoxia followed by reoxygenation may result in cellular death. Therefore an elevated ROS production in hypoxic cells may be falsified missed due to a reduced number of living cells when comparing them with normoxic control groups. To circumvent this problem we performed double live cell labeling of bEnd.3: Ahead of staining with ROS indicators cells were counterstained for 30 minutes with cell trace calcein red AM or calcein green AM at a concentration of 5 µmol/l in HBSS^+++^. Cytosolic ROS generation was quantified by staining the cells for 30 minutes using DCF at a concentration of 10 µmol/l in HBSS^+++^ as recommended by the manufacturer. Mitochondrial ROS in bEnd.3 were detected by staining the cells for 10 minutes with MitoSox at a concentration of 5 µmol/l in HBSS^+++^. DCF or MitoSOX are widely used indicators to monitor cellular (DCF) or mitochondrial ROS (MitoSox)[58]. Immediately after staining with ROS indicators cells were carefully washed once with HBSS^+++^ and fluorescence was measured in an infinite M1000 Tecan Plate reader in HBSS^+++^, data was acquired with i-control 6 software (from Tecan Trading AG, Männderdorf, Switzerland). 

Calcein red/green is converted to fluorescent dyes by intracellular esterases of living cells[59]. DCF/MitoSox signals were set in relation to calcein red/green signals. Excitation/emission wavelengths in nm for the different dyes were: 577/590 calcein red, 506/528 calcein green, 485/535 DCF, 510/580 MitoSox. Data are expressed as relative fluorescent signals. 

### In vitro transwell brain endothelial permeability assay

Changes in macromolecular permeability of bEnd.3 monolayers BEC were studied using cell culture transwell inserts (24 W 3 µm pore size, Becton Dickinson, USA). bEnd.3 were seeded at a density of 80,000 cells/well onto cell culture inserts. After bEnd.3 cells became confluent MHR and respective normoxic controls were induced as documented above. FITC-Dextran (150,000 Dalton mass weight) dissolved in HBSS^+++^ at a concentration of 1 mg/ml was added to the upper chamber 60 minutes before the end of the experiments. After incubation for 60 minutes inserts were removed from the wells and fluorescence was measured in a plate reader (infinite M1000 Tecan Plate reader, software: i-control 6 both from Tecan Trading AG, Männderdorf, Switzerland). Fluorescent excitation was 492 nm, emission was detected at 518 nm respectively. Relative fluorescent changes related to normoxic, vehicle treated controls are presented.

### Immunohistochemistry

#### bEnd.3: Nitrotyrosine detection and staining for ZO-1 and Cl-5

For immunohistochemical stainings in bEnd.3 cells were fixed for 10 minutes in ice cold acetone. Following fixation cells were washed with PBS 0.01 mol/l. Subsequently probes were blocked and permeabilized with 7 % normal goat serum and 0.3 % triton in PBS 0.01 mol/l for 2 hours at room temperature. Then primary antibodies (for detailed info please see Table **1**) were incubated over night at room temperature. Probes were washed with PBS 0.01 mol/l and secondary antibodies were incubated for another 2 h at room temperature. Cell nuclei were visualized using DAPI (Sigma, 32670 0.5 µg/ml). After a final washing step with PBS 0.01 mol/l probes were embedded in fluoromount. 

**Table 1:** Antibodies for immunohistochemistry.

*Primary antibodies (Manufacture, dilution used*)	*Secondary antibodies (Manufacture, dilution used*)	*Application, comments*
anti-zonula occludens 1 (Zymed via invitrogen, 61-300, 1:100)	Cy^TM^2-conjugated AffiniPure goat anti-rabbit IgG (H+L) , Dianova (Hamburg) 111-25-144, 1:100	Immunohistochemistry, brain endothelial cytosolic TJ linker protein
anti-claudin 5 (Zymed via invitrogen, 35-2500, 1:50)	Alexa Fluor 568 goat anti-mouse (Invitrogen Molecular Probes A11031, 1:400)	Immunohistochemistry, brain endothelial transmembraneous TJ protein
anti-nitrotyrosine (Millipore, 06-284, 1:200)	Cy3-conjugated AffiniPure Donkey anti-Rabbit IgG (H+L) 1:200 and AMCA goat anti-rabbit 1:200	Immunohistochemistry, recognizes nitrotyrosine which is formed upon reaction of tyrosine with the radical peroxynitrite
anti-MAP-2 (Neomarkers mouse monoclonal antibody, 1:1000)	biotinylated goat anti-mouse IgG (Jackson Immunoresearch Laboratories, 1:600)	MAP 2 loss is an early marker for hypoxic injury in neuronal dendrites

### Guinea Pig whole brain preparation: MAP2 and nitrotyrosine staining

For morphological studies, at the end of the experiment the brains were fixed by immersion in a cold 4% paraformaldehyde solution in phosphate buffer (PB; 0.1 M, pH 7.4). After fixation, serial coronal sections (50 mm) were cut with a vibratome (VT 1000S Leica Heidelberg, Germany).

Selected free-floating sections were incubated first for 1 h in PBS containing 10% NGS and 0.2% Triton X-100, and then overnight in the primary antibodies (against MAP 2 and or nitrotyrosine) diluted in PBS with 1% NGS at 4°C. The day after, sections were incubated for 2 h at room temperature in an indocarbocyanine (CY3)-conjugated goat–anti-mouse IgG solution (1:600, Jackson Immunoresearch Laboratories, West Grove, PA, USA) or goat-anti rabbit antibody (for details see Table **1**) and, after repeated rinsing, were mounted in Fluorsave (Calbiochem, San Diego, CA, USA). 

### Analysis of TJ impairment with linescan measurements

To analyze expression of ZO-1 and Cl5 on cell membranes in bEnd.3 cells we have previously established[50] linescan TJ analyses modified according to Brown et al.[60]. This method is now also in use by other labs for quantification of TJ alterations on cell membranes[61,62]. In brief peak fluorescence intensities at cell membranes (values V1 and V3) were related to the average intensity of the cytoplasm of each corresponding cell (distance from V1 to V3 = V2). Fiji is just ImageJ (NIH software) was used for this purpose. The following equation to calculate relative intensities of ZO-1 and Cl5 was used: [(V1 + V3) / 2] / V2. Here, 12 randomly chosen cells from 3 experiments per group were analyzed. All parameters regarding magnification, gain, laser intensity, pinhole size were carefully controlled to be the same. Linescan analysis and image acquisitions were done without background subtraction.

### Laser scanning confocal microscopy

We used a Leica TCS SP5 laser scanning confocal microscope equipped with 4 laser lines to detect immunohistochemical signals. Guinea pig probes were examined under a confocal laser scanning microscope (Bio-Rad, Hemel Hemstead, UK) equipped with an argon/krypton gas laser mounted on a light microscope (Eclipse E600; Nikon, Tokio, Japan). Excitation wavelengths were 405 nm, 488 nm and 561(Leica) / 550 (Bio-Rad) nm. Confocal image series were recorded with Bio-Rad Lasersharp 2000 software. For analysis of images Leica Application Suite Advanced Fluorescence and Fiji is just ImageJ (NIH software) were used. All parameters regarding magnification, gain, laser intensity, pinhole size were taken care of to be the same in corresponding experiments. Images without any background subtraction are displayed.

### Western Blot

For Western Blot analysis bEnd.3 cells were scraped off in 50 µl lysis buffer (containing 50 mM Tris, 150 mM NaCl, 1 mM EDTA, 1% Triton x-100 and protease and phosphatase inhibitors) and incubated 45 minutes at 4°C. After centrifugation (5000 g, 5 min, 4°C) proteins (10 µg) were separated on a 12% sodium dodecylsulfate polyacrylamide gel and blotted onto PVDF membranes. Blotting was carried out for 90 minutes at 130 V. Membranes were blocked 30 minutes in blocking buffer (4% dry milk powder in TBST) and incubated with rbαGAPDH (1:5000; Bethyl Laboratories) and mαNox2 (1:1000; Santa Cruz Biotechnology, Inc) in blocking buffer at the indicated dilutions for 1 hour. Secondary antibodies gαrbHRP (1:10000, Dianova) and gαmHRP (1:10000, Dianova) were incubated for 30 minutes.

### Statistics

Data are presented as mean values + standard error of the mean. P < 0.05 was considered to be statistically significant. All datasets were evaluated for normalization using the D’Agostino and Pearson omnibus[63] normality test. Datasets that did not pass the test for normalization were analyzed using a nonparametric Mann Whitney U test. Datasets that passed the test for normalization were analyzed using t-test. Graphpad Prism Version 4 for Windows was used for these purposes.
